# Downregulation of indoleamine-2,3-dioxygenase in cervical cancer cells suppresses tumor growth by promoting natural killer cell accumulation

**DOI:** 10.3892/or.2012.1984

**Published:** 2012-08-23

**Authors:** NAOTO SATO, YASUSHI SAGA, HIROAKI MIZUKAMI, DONGDONG WANG, SUZUYO TAKAHASHI, HIROAKI NONAKA, HIROYUKI FUJIWARA, YUJI TAKEI, SHIZUO MACHIDA, OSAMU TAKIKAWA, KEIYA OZAWA, MITSUAKI SUZUKI

**Affiliations:** 1 Department of Obstetrics and Gynecology, Center for Molecular Medicine, School of Medicine, Jichi Medical University, Tochigi; 2 Division of Genetic Therapeutics, Center for Molecular Medicine, School of Medicine, Jichi Medical University, Tochigi; 3 National Institute for Longevity Sciences, National Center for Geriatrics and Gerontology, Obu, Japan; 4 Department of Gynecology and Obstetrics, Shengjing Affiliated Hospital of China Medical University, Shenyang, P.R. China

**Keywords:** cervical cancer, indoleamine-2, 3-dioxygenase, natural killer cell, short hairpin RNA

## Abstract

This study examined the role of the immunosuppressive enzyme indoleamine-2,3-dioxygenase (IDO) in cervical cancer progression and the possible use of this enzyme for cervical cancer therapy. We analyzed IDO protein expression in 9 cervical cancer cell lines (SKG-I, -II, -IIIa, -IIIb, SiHa, CaSki, BOKU, HCS-2 and ME-180) stimulated with interferon-γ. IDO expression was observed in all cell lines except for SKG-IIIb. We transfected the human cervical cancer cell line CaSki that constitutively expresses IDO with a short hairpin RNA vector targeting IDO, and established an IDO-downregulated cell line to determine whether inhibition of IDO mediates cervical cancer progression. IDO downregulation suppressed tumor growth *in vivo*, without influencing cancer cell growth *in vitro*. Moreover, IDO downregulation enhanced the sensitivity of cervical cancer cells to natural killer (NK) cells *in vitro* and promoted NK cell accumulation in the tumor stroma *in vivo*. These findings indicate that downregulation of IDO controls cervical cancer progression by activating NK cells, suggesting IDO as a potential therapy for cervical cancer.

## Introduction

Cervical cancer is the third most common cancer in women worldwide, with global estimates of 529,800 new cases and 275100 deaths in 2008 (1). In the United States, cervical cancer is the second leading cause of cancer related deaths in young women as approximately 12,000 women were diagnosed with cervical cancer in 2010, about 4,000 of whom died from this disease (2). Although advanced cervical cancer can be treated by radical surgery with or without radiotherapy and/or chemotherapy, some patients with high risk factors will still have an unfavorable prognosis (3). The 5-year survival rate is ~70% and has not improved in the last decade (2). Therefore, new strategies, such as immunotherapy and molecular-targeted therapy, may prove useful in improving the prognosis for cervical cancer patients.

Indoleamine-2,3-dioxygenase (IDO) is an enzyme that catalyzes the first and rate-limiting step in the kynurenine pathway of tryptophan catabolism. IDO was originally discovered in 1967 (4,5) in rabbit small intestines and was purified in 1978 (6). Subsequently, it was reported that the enzyme could be induced in the mouse lung by either viral infection (7) or endotoxin shock (8). Proinflammatory mediators, such as interferon or other cytokines, can also stimulate IDO induction (9). A study revealing that IDO in the mouse placenta prevented rejection of the allogeneic fetus exposed its immunosuppressive properties (10). Recently, it was demonstrated that IDO can induce immunotolerance in patients with autoimmune diseases (11) and chronic infections (12). Most human malignant tumors express IDO (13), and IDO can contribute to tumor-induced immunosuppression by starving T-cells, which are sensitive to tryptophan deficiency. In this environment, tumor cells can escape immune surveillance via the action of IDO (10).

Natural killer (NK) cells are important members of the innate immune system, which plays a role in inhibiting the growth of several types of tumors (14). Tryptophan-derived catabolic kynurenine can reduce NK cell number and weaken NK cell cytotoxicity by inhibiting the expression of NK cell receptors, thus contributing to tumor progression (15). In gynecology, IDO expression has been observed in cervical, endometrial and ovarian cancers (13), and associations between IDO expression and prognosis of in these cancers have been reported (16–19).

RNA interference (RNAi) is a technique for gene silencing and involves a post-transcriptional gene-silencing mechanism (20). Among the different types of RNAi techniques, the use of small interfering RNAs (siRNAs) effectively suppresses gene expression in a transient manner (21). Short hairpin RNAs (shRNAs) driven by polymerase III promoters have been developed to attain long-term stable target gene silencing (22,23).

In this study, we used an shRNA vector to silence IDO expression in an IDO-expressing cervical cancer cell line to further elucidate the relationship between expression and cervical cancer growth. Moreover, we investigated the function of NK cells in cervical cancer progression in order to develop an IDO-targeted molecular therapy for cervical cancer.

## Materials and methods

### Cell culture

The 9 cervical cancer cell lines used in this study (SKG-I, -II, -IIIa, -IIIb, SiHa, CaSki, BOKU, HCS-2 and ME-180) (24–30) were obtained as follows: the SKG-I, -II, -IIIa and IIIb lines were obtained from Dr Daisuke Aoki (Keio University, Tokyo, Japan); the SiHa line was purchased from the American Type Culture Collection (ATCC, Manassas, VA); and the CaSki, BOKU, HCS-2 and ME-180, were all purchased from the Japanese Collection of Research Bioresources (JCRB, Osaka, Japan). These cell lines were maintained in D-MEM/Ham’s F-12 medium (DMEM/F12, Gibco, Grand Island, NY) containing 10% inactivated fetal calf serum (Sigma, St. Louis, MO), 100 U/ml penicillin (Gibco) and 100 μg/ml streptomycin (Gibco) at 37°C in a 5% CO_2_ atmosphere for no longer than 8 weeks after recovery from frozen stocks.

The NK cell line KHYG-1 (31) was purchased from the JCRB. Cells were cultured in RPMI-1640 medium supplemented with 100 nM of human interleukin-2 (R&D Systems, Minneapolis, MN) and 10% inactivated fetal calf serum (Sigma), at 37°C in a 5% CO_2_ atmosphere for no longer than 8 weeks after recovery from frozen stocks.

### Antibodies

The anti-human IDO monoclonal antibody was prepared as previously reported (32). Anti-human actin (SIGMA) and anti-mouse CD49b antibodies (R&D Systems) were used according to manufacturer’s protocols.

### shRNA stable cell line and control cell line

The short hairpin RNA (shRNA) plasmid targeting IDO gene expression (piGENE PURhU6/shIDO) and control plasmid (piGENE PURhU6) have been previously described (33), and were transfected into the CaSki cell line using Lipofectamine LTX and Plus Reagent (Invitrogen, Carlsbad, CA) according to the manufacturer’s instructions. Transfected cells were selected for using 0.5 μg/ml puromycin (Calbiochem, Darmstadt, Germany). Resistant CaSki/shIDO and CaSki/Mock clones were obtained after 4 weeks. The cells were subsequently maintained in the presence of 0.5 μg/ml puromycin.

### Western blot analysis

Before protein extraction for western blot analysis, cervical cancer cells were cultured in DMEM/F12 with 100 ng/ml interferon-γ (R&D Systems) for 24 h. Ten micrograms of protein extracted from a cultured cell homogenate was mixed with 2X SDS-PAGE sample buffer [120 mM Tris-HCl (pH 6.8), 4% SDS, 20% glycerol, 0.004% bromophenol blue and 10% 2-mercaptoethanol]. The mixture was heated at 95°C for 2 min, and electrophoresed on a 0.1% SDS-10% polyacrylamide gel, before blotting the proteins onto a polyfluorovinylidene membrane. The membrane were blocked with a Non-Protein Blocking Agent (ATTO Corp., Tokyo, Japan) at room temperature for 1 h, and incubated with the anti-human IDO monoclonal (1:1000) and anti-human actin polyclonal antibodies (1:200) for 1 h at room temperature. The membrane was washed with phosphate-buffered saline (PBS)-Tween-20 3 times and then incubated with a horseradish peroxidase-conjugated secondary anti-mouse (Thermo, Rockford, IL) or anti-rabbit antibody (Thermo). Signals were detected by chemiluminescence (ECL kit; Amersham Biosciences, Piscataway, NJ) on X-ray film.

### In vitro cell growth kinetics

Five-hundred CaSki/shIDO and CaSki/Mock cells were seeded onto a 96-well plate and cultured in DMEM/F12 medium containing 10% fetal calf serum. Every 24 h, cells were counted using a colorimetric assay with the Cell Proliferation kit II (XTT) (Boehringer Mannheim GmbH Biochemica, Mannheim, Germany) and a growth curve was drawn from the results.

### Sensitivity of transfectants to NK cells in vitro

The sensitivity of CaSki/shIDO and CaSki/Mock cells to NK cells was investigated by colorimetric assay using XTT. Five-hundred CaSki/shIDO and CaSki/Mock cells were seeded onto a 96-well plate and co-cultured with KHYG-1 cells (0, 500, 1000, 2000 or 4000 cells) in DMEM/F12 medium containing 10% fetal calf serum for 72 h. After 3 washes with PBS to completely remove KHYG-1 cells, viable cell count was determined by colorimetric assay and calculated as the percent of control cells (respective cell lines cultured without KHYG-1 cells).

### Experimental animals

Four- to six-week-old female BALB/c nude mice (Japan Clea Laboratories, Tokyo, Japan) were used in this study. All animal experiments were conducted according to the institutional and national guidelines for animal experiments.

### Subcutaneous tumor growth in vivo

CaSki/shIDO and CaSki/Mock cells (5×10^6^ cells from each line) were inoculated subcutaneously into the back of mice to induce tumor growth. The tumor volume [(long diameter) × (short diameter)^2^ × 1/2] was measured twice a week to generate a tumor growth curve.

### Immunohistochemical staining

One week after subcutaneous tumor cell inoculation, mice were sacrificed under isoflurane anesthesia, and the tumor was removed. After formalin fixation, paraffin sections were prepared, deparaffinized, and treated with hydrogen peroxide for 30 min to block endogenous peroxidase. The sections were then reacted with a 1:10 dilution (5 μg/ml) of anti-mouse CD49b primary antibody for 16 h at room temperature, washed 3 times washes with PBS, and then incubated with enzyme-conjugated streptavidin for 30 min. The sections were again washed with PBS 3 times, and color was developed using the diaminobenzidine method. The number of stained NK cells was counted under high-power magnification (×400).

### Statistical analysis

The test of significance between the 2 groups was performed using Student’s t-test. A P-value of <0.05 was considered significant.

## Results

### IDO expression

**Figure 1:**
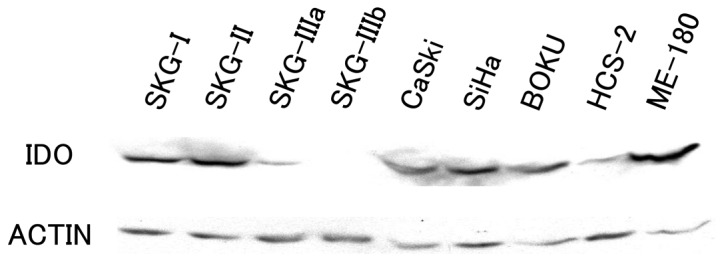
Western blotting using an anti-IDO monoclonal antibody. IDO expression was detected at the position corresponding to a molecular weight of 41 kDa in all cell lines except for the SKG-IIIb line.

As shown in Fig. 1, IDO expression was detected by western blotting at a position corresponding to a molecular weight of 41 kDa in all cell lines except for the SKG-IIIb line.

### Establishing an IDO-downregulated cell line

**Figure 2:**
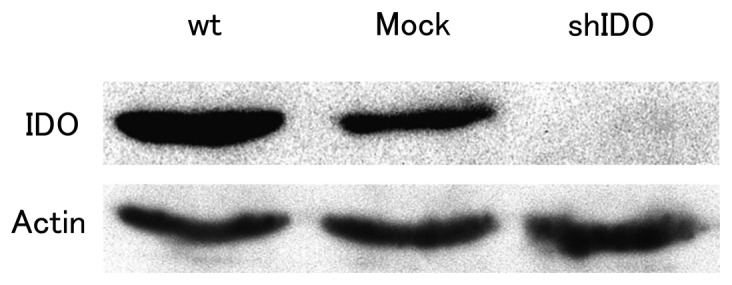
Western blot analysis of parental cells (wt) and control vector-transfected cells (Mock) showing IDO expression. In contrast, the shIDO vector-transfected cells (shIDO) did not show IDO expression.

Fig. 2 shows the results of western blot analysis of the CaSki cervical cancer cell line transfected with either an shIDO expression vector or a control vector. Parental cells (wt) and control vector-transfected cells (Mock) expressed IDO. In contrast, the shIDO expression vector-transfected cells (shIDO) did not show IDO expression, confirming IDO downregulation in the CaSki/shIDO cell line.

### In vitro cell growth kinetics

**Figure 3:**
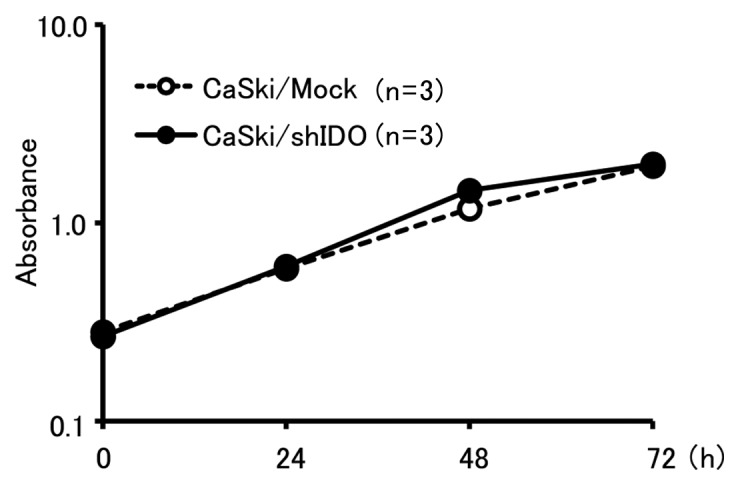
Cell growth curves of CaSki/shIDO and CaSki/Mock (control) cells. There was no significant difference between the 2 groups. Results are expressed as means ± SD.

Growth curve analyses of CaSki/shIDO and CaSki/Mock cells revealed no significant differences between the two groups, suggesting that IDO downregulation did not affect cell growth *in vitro* (Fig. 3).

### Sensitivity of transfectants to NK cells in vitro

**Figure 4:**
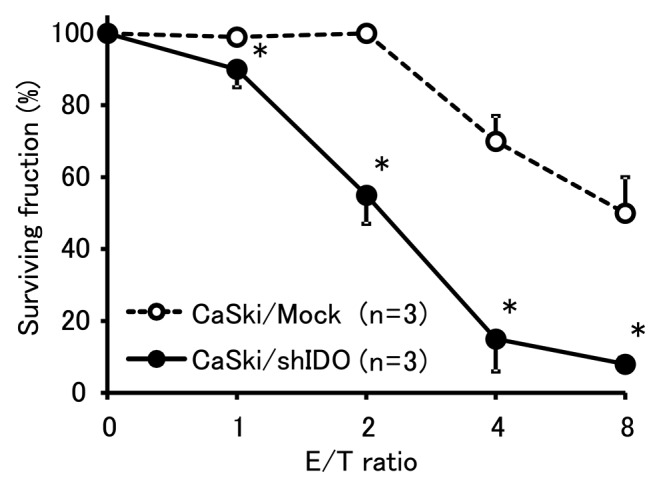
The percentage of viable tumor cells co-cultured with NK cells. The percent survival of CaSki/shIDO cells was significantly lower than that of control cells. ^*^P<0.01. The results are expressed as means ± SD.

The proportion of viable tumor cells co-cultured with NK cells is shown in Fig. 4. The percent survival of CaSki/shIDO cells was significantly lower than that of the control cells, indicating that the downregulation of IDO increased the sensitivity of tumor cells to NK cells.

### Tumor growth in vivo

**Figure 5:**
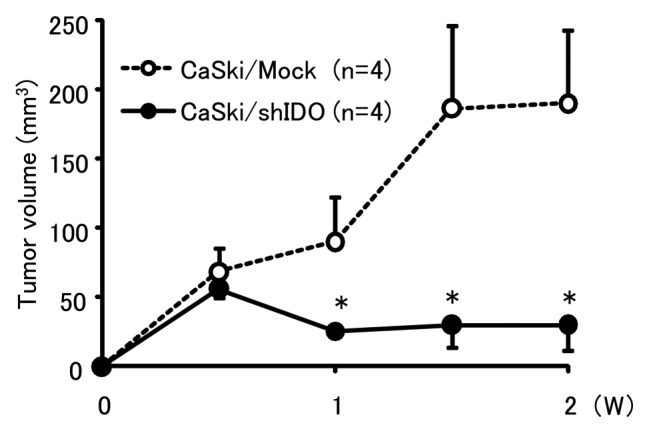
Subcutaneous tumor growth curves of CaSki/shIDO and control cells. Both groups of cells formed small nodules 3 days after inoculation. Subsequently, the tumors in the control group enlarged, whereas those in the CaSki/shIDO group disappeared. ^*^P<0.05; mean ± SD.

Both CaSki/shIDO and control cells formed small nodules 3 days after inoculation (Fig. 5). Subsequently, the tumors in the control group enlarged, whereas those in the CaSki/shIDO group reduced in size, suggesting that the downregulation of IDO inhibited tumor growth *in vivo*.

### Number of NK cells in the tumor stroma

**Figure 6:**
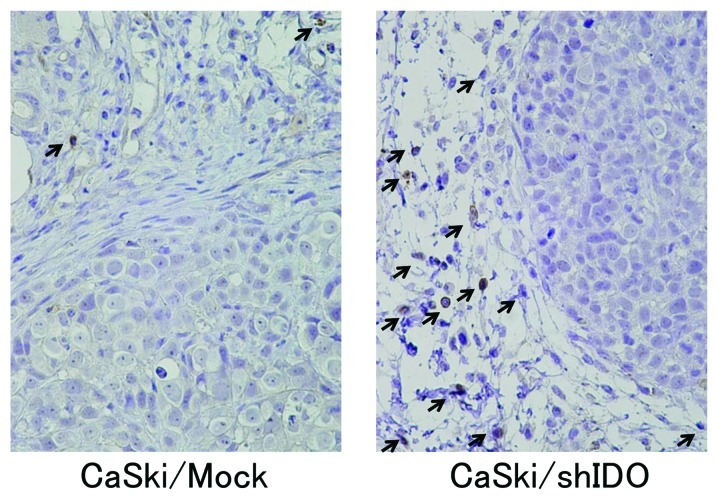
CD49b expression in CaSki/shIDO and control subcutaneous tumors. The black arrows indicate NK cells accumulating in the tumor stroma.

NK cells immunostaining (black arrows) reveals an accumulation of NK cells in the stroma of both CaSki/shIDO and control subcutaneous tumors (Fig. 6). The number of NK cells (24±8) that accumulated in CaSki/shIDO tumors was significantly higher than (2±2) in the control tumors (P<0.01). These results suggest that the downregulation of IDO promoted NK cell accumulation around the tumor.

## Discussion

The experiments described in this study aim to clarify the relationship between the immunosuppressive enzyme IDO and cervical cancer progression, as well as to develop a molecular therapy targeting IDO. First, we investigated the expression of IDO in 9 cervical cancer cell lines stimulated with interferon-γ and observed that all of them, except the SKG-IIIb line, expressed IDO. These results suggest that many cervical cancer cells produce IDO. Next, we utilized an shRNA expression vector targeting the IDO gene to examine whether the inhibition of IDO can control cervical cancer tumor growth. We found that the downregulation of IDO expression did not influence cervical cancer cell growth *in vitro*, but controlled tumor growth *in vivo*. In addition, the downregulation of IDO increased the sensitivity of cervical cancer cells to NK cells *in vitro* and promoted NK cell accumulation in the tumor stroma *in vivo*. These findings indicate that downregulation of IDO effects cervical cancer tumor growth by promoting NK cell accumulation in tumors, suggesting that IDO may be a useful therapeutic target for patients with cervical cancer.

There are a few reports that describe IDO expression in cervical cancer. Inaba *et al* reported that IDO is expressed in 52% of invasive cervical cancer cases as determined by immunohistochemical staining (16). On the other hand, Nakamura *et al* reported that IDO expression was detected in all 25 cases of invasive cervical cancer (34). In addition, both reports observed that IDO expression in invasive cancer was confined to the cancer cells at the invasive front (16,34). Since these cells are in an environment where they are easily exposed to proinflammatory mediators, such as interferon-γ or other cytokines, they may be stimulated to produce IDO. In our study, although only 2 of 9 cervical cancer cell lines constitutively expressed IDO (CaSki and BOKU, data not shown), all cell lines except for SKG-IIIb cells expressed IDO after stimulation with interferon-γ. These results suggest that many cervical cancer cells have the capability to produce IDO.

The lack of the essential amino acid tryptophan and accumulation of its metabolite, kynurenine, inhibit cell growth and induce cell death. T-cells are particularly sensitive to this type of stress (10). Regarding the mechanism of cancer cell immunotolerance, IDO has been shown to promote local tryptophan depletion, resulting in T-cell function inhibition in the vicinity of IDO-expressing cancer cells and general local immunotolerance (13). The possibility that IDO expression is involved in the immunotolerance of cervical cancer through such a T-cell mediated mechanism cannot be excluded. However, a cervical cancer cell line that can form a tumor in immunocompetent mice has not been found. Therefore, we chose the human cervical cancer cell line (CaSki) that constitutively expresses IDO and implanted this cell line in nude mice. Since nude mice congenitally lack T cells, in this experimental system, we were not able to examine the effect of IDO on T-cell function.

It has been reported that IDO promotes the accumulation of the tryptophan metabolite kynurenine, which suppresses the expression of NK cell receptors, and thereby inhibits the NK cell function (15). Similarly, in our previous experiments using ovarian cancer cells, IDO expression inhibited the cytotoxic activity of NK cells *in vitro* and suppressed NK cell accumulation in the tumor stroma *in vivo*(35). Here, we demonstrated that IDO downregulation enhanced the sensitivity of cervical cancer cells to NK cells *in vitro* and promoted NK cell accumulation in the cervical cancer stroma *in vivo*. Thus, IDO downregulation reinforced the sensitivity of cancer cells to NK cells and suppressed cervical cancer growth.

To date, chemically synthesized siRNA and vector-mediated expression of shRNA are the most commonly used RNAi gene silencing techniques (36,37). Although siRNA can be more easily transfected into mammalian cells and its silencing ability is more effective than shRNA, its effects are transient. The remarkable advantage of shRNA is that the inhibition of target genes can last for weeks or even months, making it possible to elucidate the consequences of long-term, stable gene silencing (36). In actual clinical settings, viral-based expression vectors or nanoparticle-based vectors (38) could be used to deliver IDO shRNA to the cancer cells.

The results of this study demonstrate that the downregulation of IDO in human cervical cancer cells that constitutively express this enzyme inhibits cervical cancer progression. This suggests that IDO-targeted shRNA is a potentially effective molecular-targeted therapy for cervical cancer.
